# Information Geometry and Manifold Learning: A Novel Framework for Analyzing Alzheimer’s Disease MRI Data

**DOI:** 10.3390/diagnostics15020153

**Published:** 2025-01-10

**Authors:** Ömer Akgüller, Mehmet Ali Balcı, Gabriela Cioca

**Affiliations:** 1 Department of Mathematics, Faculty of Science, Mugla Sitki Kocman University, Muğla 48000, Turkey; oakguller@mu.edu.tr; 2 Preclinical Department, Faculty of Medicine, Lucian Blaga University of Sibiu, 550024 Sibiu, Romania; gabriela.cioca@ulbsibiu.ro

**Keywords:** information geometry, statistical manifold, manifold learning, impairment classes

## Abstract

**Background**: Alzheimer’s disease is a progressive neurological condition marked by a decline in cognitive abilities. Early diagnosis is crucial but challenging due to overlapping symptoms among impairment stages, necessitating non-invasive, reliable diagnostic tools. **Methods**: We applied information geometry and manifold learning to analyze grayscale MRI scans classified into No Impairment, Very Mild, Mild, and Moderate Impairment. Preprocessed images were reduced via Principal Component Analysis (retaining 95% variance) and converted into statistical manifolds using estimated mean vectors and covariance matrices. Geodesic distances, computed with the Fisher Information metric, quantified class differences. Graph Neural Networks, including Graph Convolutional Networks (GCN), Graph Attention Networks (GAT), and GraphSAGE, were utilized to categorize impairment levels using graph-based representations of the MRI data. **Results**: Significant differences in covariance structures were observed, with increased variability and stronger feature correlations at higher impairment levels. Geodesic distances between No Impairment and Mild Impairment (58.68, p<0.001) and between Mild and Moderate Impairment (58.28, p<0.001) are statistically significant. GCN and GraphSAGE achieve perfect classification accuracy (precision, recall, F1-Score: 1.0), correctly identifying all instances across classes. GAT attains an overall accuracy of 59.61%, with variable performance across classes. **Conclusions**: Integrating information geometry, manifold learning, and GNNs effectively differentiates AD impairment stages from MRI data. The strong performance of GCN and GraphSAGE indicates their potential to assist clinicians in the early identification and tracking of Alzheimer’s disease progression.

## 1. Introduction

Alzheimer’s disease is a progressive neurodegenerative disorder characterized by the gradual decline of cognitive functions, including memory, reasoning, and the ability to perform everyday activities. This debilitating condition not only affects the individuals diagnosed but also has profound implications for their families, caregivers, and the broader healthcare system. Recent data indicates that more than 50 million individuals globally are affected by dementia, with Alzheimer’s disease comprising roughly 60–70% of these diagnoses [1]. The prevalence of Alzheimer’s disease is projected to rise substantially with the aging global population, potentially tripling by 2050 [2,3]. This anticipated surge presents significant challenges, including increased emotional and financial burdens on families, heightened stress levels for caregivers, and substantial strains on healthcare systems. The need for resources to support long-term care and medical interventions will intensify, necessitating advancements in diagnostic tools and treatment strategies to manage this growing public health concern effectively.

Identifying Alzheimer’s disease early and accurately is essential for establishing effective treatment and care strategies. Detecting the condition promptly enables actions that can hinder its advancement, alleviate symptoms, and improve the well-being of both individuals affected and their relatives. Moreover, early diagnosis enables patients to participate in clinical trials, access experimental therapies, and make informed decisions regarding their future care and living arrangements [4,5]. Detecting the disease in its early stages enables clinicians to develop personalized treatment plans that may help slow cognitive and functional decline, prolonging the independence of the patient and lessening its impact on daily life.

Accurately diagnosing Alzheimer’s disease, particularly in its early stages, remains a significant challenge. Current methods typically rely on a combination of clinical evaluations, neuropsychological testing, and biomarker analysis [6,7]. These diagnostic techniques often involve invasive procedures, such as lumbar punctures for cerebrospinal fluid examination or PET scans with radioactive tracers, which are expensive and pose potential risks to patients. Additionally, these methods may lack the accuracy and precision required to reliably distinguish between normal aging, mild cognitive impairment (MCI), and the early stages of Alzheimer’s disease [8]. The overlap of symptoms and subtle brain changes during the initial phases of the disease further complicates accurate diagnosis. Therefore, there is an urgent need for reliable, non-invasive diagnostic techniques capable of accurately identifying and classifying the stages of cognitive decline associated with Alzheimer’s disease, facilitating earlier and more effective interventions.

Magnetic Resonance Imaging (MRI) has become a highly effective and non-intrusive technique for assessing the structural health of brain tissues. MRI delivers comprehensive visuals of brain anatomy, enabling healthcare professionals and scientists to identify structural alterations such as cortical thinning, hippocampal shrinkage, and modifications in white matter integrity, which are key indicators of Alzheimer’s disease [9,10]. Structural alterations may develop several years before clinical symptoms appear, making MRI an essential tool for the early identification and tracking of Alzheimer’s disease progression. However, analyzing high-dimensional MRI data is highly challenging due to its immense complexity and large volume. Conventional analysis techniques, which often depend on manual or partially automated evaluations, might not fully detect the intricate and subtle morphological patterns that indicate disease advancement. The extensive dimensionality of MRI data requires sophisticated analytical methods capable of uncovering meaningful patterns and relationships, therefore enhancing diagnostic accuracy and providing deeper insights into the neurodegenerative processes associated with Alzheimer’s disease.

Machine learning offers promising solutions for effectively managing and interpreting high-dimensional medical imaging data. Conventional machine-learning techniques, such as support vector machines (SVMs) and random forests, have been utilized to categorize MRI data for the diagnosis of Alzheimer’s disease [11,12,13,14]. These methods typically involve the extraction of handcrafted features, such as volumetric measurements of specific brain regions or texture features that quantify tissue characteristics. While these approaches can achieve reasonable levels of accuracy, they often require significant domain expertise for feature selection and may struggle to scale with the increasing complexity and volume of data. Conversely, deep-learning approaches, especially convolutional neural networks (CNNs), have transformed image classification tasks by autonomously deriving layered feature representations directly from unprocessed data [15,16,17]. Convolutional neural networks have exhibited exceptional effectiveness in identifying Alzheimer’s disease–associated modifications in MRI scans, reaching high levels of accuracy in differentiating between individuals with and without the condition [18,19,20,21]. Nevertheless, deep-learning algorithms generally require extensive labeled datasets and considerable computational power for training. This poses challenges in medical settings where data availability is limited and computational resources are restricted.

Information geometry offers a powerful mathematical framework for examining the geometric structures of statistical models, providing valuable insights into patterns and relationships within high-dimensional data [22]. By representing probability distributions as points on a statistical manifold, information geometry facilitates the analysis of complex and nonlinear data relationships. Manifold learning methods, such as Isomap, locally linear embedding (LLE), and t-distributed stochastic neighbor embedding (t-SNE), complement this framework by uncovering the underlying geometric structure of data, enabling the identification of nonlinear patterns that traditional linear methods might miss [23,24,25,26]. These techniques hold significant promise for modeling the progression of Alzheimer’s disease, as they can detect and quantify subtle structural changes in the brain observed in MRI scans. By understanding these geometric transformations associated with cognitive decline, these methods can contribute to the development of more accurate and reliable diagnostic tools.

Graph Neural Networks (GNNs) have emerged as a highly effective class of models capable of leveraging graph-structured data to capture complex relational information [27,28]. GNNs extend traditional neural networks by incorporating connections between data points, allowing them to represent intricate interactions and dependencies often present in real-world datasets. These models have achieved notable success across various domains, including bioinformatics, social network analysis, and natural language processing [29,30,31,32,33]. In medical imaging, GNNs have been applied to map connections between brain regions or subjects, providing a more detailed and holistic representation of the data [34,35,36]. By capturing both local and global relationships within the data, GNNs enhance the accuracy and reliability of classifying and predicting levels of cognitive impairment.

Combining information geometry, manifold learning, and GNNs offers an innovative strategy for diagnosing Alzheimer’s disease. This approach involves carefully building statistical manifolds from high-dimensional MRI data and then transforming these manifolds into graph structures. Such a method effectively captures both the detailed geometric configurations and the intricate relational dynamics present in the data. Information geometry supplies a solid mathematical foundation for analyzing the fundamental statistical characteristics of the brain’s anatomical features, enabling accurate identification of variations and patterns linked to various stages of cognitive decline. At the same time, manifold learning techniques successfully decrease the dimensionality of MRI data, maintaining crucial structural details while emphasizing the nonlinear relationships that conventional linear methods might miss. Reducing dimensionality is essential for handling the extensive complexity of MRI datasets, ensuring that further analyses remain computationally manageable and statistically robust.

This study addresses a critical gap in current research by integrating information geometry, manifold learning, and GNNs to classify impairment levels in Alzheimer’s disease. The research focuses on three key objectives: first, to uncover and analyze the geometric relationships inherent in high-dimensional MRI data using information geometry and manifold learning; second, to utilize GNNs to model and interpret the relational information between data points on the statistical manifold; therefore improving the representation of interconnected brain regions; and third, to achieve highly accurate classification of cognitive impairment stages, crucial for early detection and ongoing monitoring of Alzheimer’s disease. By combining the strengths of these methodologies, this approach aims to provide a comprehensive framework that overcomes the limitations of individual techniques.

The novelty of our approach is rooted in the seamless combination of these advanced analytical methods to confront the multifaceted challenges inherent in Alzheimer’s disease diagnosis. Traditional diagnostic tools often fall short in distinguishing between subtle stages of cognitive decline due to their reliance on linear and isolated feature analyses. In contrast, our integrated approach harnesses the geometric insights provided by information geometry to understand the global structure of brain data, while manifold learning ensures that the intrinsic dimensionality and nonlinear relationships are faithfully represented. GNNs further enhance this framework by effectively modeling the complex interdependencies between different brain regions, as captured by the statistical manifolds. This holistic integration not only improves the accuracy of classification but also provides a deeper, more nuanced understanding of the disease’s progression. By amalgamating geometric and relational information, our approach aspires to develop a more sophisticated and effective diagnostic tool. Such a tool holds the promise of significantly aiding clinicians in the early detection of Alzheimer’s disease, facilitating timely interventions, and ultimately contributing to improved patient outcomes through more personalized and targeted treatment strategies.

The remainder of this paper is organized as follows: Section 2 reviews the existing literature related to Alzheimer’s disease diagnosis, encompassing traditional machine-learning methods, deep-learning advancements, manifold learning techniques, and the application of GNNs in medical imaging. Section 3 outlines the methodology employed in this study, with subsections dedicated to information geometry and manifold learning, detailing the theoretical foundations and practical implementations of these approaches. In Section 4, we present the results, beginning with an overview of the dataset used, followed by the analysis of information geometric results and the outcomes of manifold learning. In Section 5, we present detailed discussions on the results. Finally, Section 6 concludes the paper by summarizing the main findings, addressing the implications of our research, acknowledging the limitations, and proposing directions for future work to further enhance the diagnosis and classification of Alzheimer’s disease.

## 2. Related Works

Conventional machine-learning techniques have been extensively utilized for diagnosing Alzheimer’s disease using MRI data. Early studies employed methods such as SVMs, k-nearest neighbors, and logistic regression to classify patients based on features like cortical thickness, hippocampal volume, and texture metrics. Ref. [37] proposed an MRI-based biomarker combining cortical thickness, hippocampal shape, and texture, achieving a classification accuracy of 62.7% in distinguishing Alzheimer’s disease, mild cognitive impairment (MCI), and healthy controls. These features significantly contributed to diagnostic performance. Ref. [38] highlighted hippocampal volumetric reduction as a key marker of Alzheimer’s disease, computed using semi-automatic segmentation and analyzed with machine-learning techniques. Their approach demonstrated the ability of brain MRI data to differentiate between Alzheimer’s disease, MCI, and cognitively normal individuals, enhancing computer-aided diagnostic systems. Ref. [39] found that hippocampal texture analysis outperformed volumetric measurements in predicting MCI progression to Alzheimer’s disease, revealing a stronger correlation with cognitive decline. Ref. [40] applied sparse coding to cortical thickness analysis, improving SVM classification accuracy for distinguishing cognitively unimpaired individuals, MCI, and Alzheimer’s groups. Ref. [41] integrated cortical thickness, hippocampal volume, and other anatomical MRI features using elastic net logistic regression, achieving a diagnostic accuracy of 98% in distinguishing Alzheimer’s disease from control subjects. Similarly, Ref. [42] employed multi-kernel SVMs to incorporate cortical morphological patterns, achieving classification accuracies of 92.35% for Alzheimer’s diagnosis and 83.75% for MCI classification. These approaches relied on manually engineered features to capture structural changes associated with Alzheimer’s disease. While they achieved notable accuracy levels, they often required extensive domain knowledge and significant manual effort for feature selection and extraction.

The advent of deep learning has profoundly influenced the field of medical imaging, particularly in Alzheimer’s disease diagnosis. CNNs have been widely adopted for their ability to autonomously learn hierarchical feature representations directly from raw MRI scans. Ref. [43] employed the Xception architecture for MRI classification, achieving an impressive 99.6% accuracy in distinguishing various stages of Alzheimer’s disease, underscoring the potential of CNNs for precise and early diagnosis. Ref. [44] introduced the Alzheimer’s Disease Detection Network, a novel CNN-based architecture that effectively addresses class imbalance and achieves superior performance across metrics such as accuracy, precision, recall, F1-Score, and AUC on MRI datasets, outperforming existing state-of-the-art methods. Ref. [45] leveraged ResNet50 for automatic feature extraction and diagnosis, attaining accuracies of up to 99%, highlighting the advantages of CNNs in achieving both high accuracy and automation compared to traditional techniques. Ref. [46] applied unsupervised CNNs to distinguish between mild cognitive impairment and Alzheimer’s disease, achieving accuracies of up to 97.01%. This demonstrates the versatility of CNNs in automating feature extraction processes, making them highly effective for Alzheimer’s disease diagnosis. CNNs have consistently outperformed traditional methods, delivering higher accuracy in distinguishing Alzheimer’s patients from healthy controls. However, deep-learning models often require extensive labeled datasets and significant computational resources, posing challenges in medical imaging. The limited availability of labeled data and the high dimensionality of MRI scans can hinder the effective training of CNN models.

Manifold learning techniques aim to address these challenges by reducing the dimensionality of high-dimensional datasets while preserving their intrinsic geometric structure. Ref. [47] highlights the capabilities of ManifoldNet, a novel deep neural network framework tailored for manifold-valued data, which excels in tasks such as predicting Parkinson’s disease (PD) clinical scores and identifying group differences in brain connectivity. By leveraging weighted Fréchet Mean layers, this framework achieves superior efficiency and performance compared to traditional approaches. Ref. [48] provides a comparative review of linear and nonlinear neural manifold learning methods, including PCA, t-SNE, and UMAP. These techniques are shown to effectively uncover low-dimensional representations of neural activity. Applied to datasets from hippocampal place cells, motor cortical neurons, and prefrontal neurons, the study demonstrates that nonlinear methods often reveal lower-dimensional manifolds in complex behavioral tasks. Furthermore, it emphasizes the potential of neural manifold analysis for studying neurological disorders, as illustrated by simulations of Alzheimer’s disease, offering insights into circuit-level neuropathology. Ref. [49] investigates Alzheimer’s disease progression using manifold learning methods such as Spectral Embedding, Isomap, t-SNE, and UMAP on the ADNI dataset. These approaches reveal lower-dimensional embeddings that effectively differentiate early-stage AD categories. The findings also identify statistically significant sub-groupings within existing Alzheimer’s disease categories, particularly during transitions of mild cognitive impairment, underscoring the need for more granular subcategories to better characterize Alzheimer’s disease progression. Ref. [50] demonstrates that kernel-based nonlinear manifold learning is effective in detecting functional connectivity changes within EEG data. This method reveals significant differences in occipital and fronto-parietal regions, crucial for diagnosing Alzheimer’s disease, consistent with prior neuroimaging studies. Ref. [51] presents a prognostic model that uses functional connectivity gradients and penalized Cox regression to predict the likelihood of progression from mild cognitive impairment to Alzheimer’s disease. Key contributors, including the heteromodal association cortex, visual cortex, caudate, and hippocampus, are identified, with validation confirming the model’s clinical relevance for early intervention. While these methods provide valuable insights, they are largely exploratory and may not directly improve classification performance.

Information geometry offers a robust theoretical framework for analyzing the geometric properties of statistical models. By representing probability distributions as points on a manifold, it facilitates the exploration of complex data structures using concepts like geodesic distances and curvature. In medical imaging, information geometry has been applied to model variability in brain activity and analyze functional connectivity networks. Ref. [52] introduces an information geometry-based approach to analyze simulated EEG signals, revealing that healthy individuals exhibit directional changes in net causal connectivities between brain regions when transitioning between eyes-closed and eyes-open conditions. This distinction is absent in Alzheimer’s disease patients, highlighting the potential of these measures for understanding neural information processing and diagnosing neurological disorders. Ref. [53] compares four geometric deep-learning approaches utilizing hippocampal mesh representations for Alzheimer’s disease prediction. The study finds that template-based methods outperform template-free approaches in terms of accuracy, efficiency, and training speed while noting the increased preprocessing complexity associated with template-based methods and their potential to design more robust neuroimaging architectures. Ref. [54] proposes a neurofunctional framework for consciousness, demonstrating that disruptions caused by pharmacological, neuropathological, or psychiatric factors degrade cortical gradients. The study links these state-dependent reconfigurations to behavioral unresponsiveness and temporal disruptions of dynamic brain states. Ref. [55] investigates the decomposition of synergistic and redundant information to deepen our understanding of integrative brain function. This research highlights the structural, molecular, and functional roles of this decomposition in cognition and computation, proposing it as a fundamental principle for understanding the informational architecture of the brain. Applying information geometry to MRI data enables the detection of subtle morphological changes that indicate disease progression, offering a valuable tool for studying and diagnosing neurodegenerative conditions like Alzheimer’s disease.

Graph-based methodologies have gained prominence in neuroimaging for modeling the structural and functional networks of the brain. In such graphs, nodes represent distinct brain regions, while edges indicate connections based on anatomical proximity or functional correlations. Ref. [56] presents two graph-based convolutional neural network models that leverage structural and functional brain connectivity for EEG motor imagery classification. These models achieve state-of-the-art accuracy (Adj-CNNM: 72.77%, PLV-CNNM: 75.10%) and provide valuable insights into connectivity patterns, such as strong interactions between the frontal, central, and parietal cortices, therefore enhancing our understanding of brain function and its potential in neurological research. Ref. [57] highlights how Alzheimer’s disease alters microstructural patterns and morphological similarity, particularly in the insula and hippocampus. Additionally, the study observes changes in structural randomness within the temporal, frontal, and occipital regions, which correlate with cognitive performance. These findings provide critical insights into the structural organization of the brain in the context of Alzheimer’s disease. Ref. [58] demonstrates that utilizing Network Control Theory to identify personalized brain stimulation targets significantly enhances network engagement compared to conventional neuromodulation sites. This underscores the potential of customized, connectivity-based approaches in improving the efficacy of brain stimulation interventions.

Graph Neural Networks (GNNs) extend traditional neural networks to process graph-structured data, making them well-suited for various neuroscience applications, including brain network classification and disease prediction. Ref. [59] introduces the Directed Structure Learning GNN (DSL-GNN) framework, which integrates effective brain connectivity and power spectrum density features to classify Alzheimer’s disease (AD), Parkinson’s disease (PD) and healthy controls with high accuracy, reaching up to 97.4% for AD vs. PD classification. This highlights DSL-GNN’s potential as a powerful tool for diagnosing neurodegenerative diseases by analyzing complex brain networks. Ref. [60] proposes a heterogeneous graph neural network (FC-HGNN) for analyzing brain functional connectivity. By combining individual and population graph approaches, FC-HGNN achieves state-of-the-art accuracy in classification and biomarker identification for mental disorder diagnosis. The model’s validation on public datasets and clinical data addresses challenges in both interpretability and predictive performance. Ref. [61] introduces a multimodal graph convolutional network capable of simultaneously processing temporal and spatial fMRI features along with demographic data. This model achieves an 80.66% classification accuracy, outperforming traditional graph neural networks by 10%. It also uncovers novel autism-related biomarkers, validated by existing literature, demonstrating its potential to advance brain disorder diagnosis and research. Ref. [62] presents the Granger Causality-Inspired Graph Neural Network (CI-GNN), an interpretable model that distinguishes between causal and non-causal subgraph representations. This approach identifies functionally connected brain regions relevant to psychiatric diagnoses. By leveraging conditional mutual information for regularization, CI-GNN avoids spurious correlations, outperforms baseline GNNs and explainers across various metrics, and delivers clinically validated, reliable explanations.

Despite the promising advancements in geometric and graph-based methodologies for Alzheimer’s disease diagnosis, several significant limitations persist. Geometric approaches, such as those leveraging information geometry, often face challenges related to the high computational complexity associated with modeling complex brain structures, which can impede scalability to larger datasets [63]. Additionally, these methods may struggle with capturing nonlinear relationships and intricate interactions between multiple brain regions, potentially limiting their ability to fully represent the underlying pathological processes [64]. On the other hand, graph-based approaches, including GNNs, while effective in modeling brain connectivity, are frequently constrained by the quality and completeness of the predefined brain networks [65]. They can also be sensitive to noise and variability in neuroimaging data, leading to reduced robustness and generalizability across different populations and imaging protocols [66,67]. Furthermore, many existing graph-based models lack interpretability, making it difficult to elucidate the specific neurobiological mechanisms driving their diagnostic predictions [68]. Another critical limitation is the limited integration of multimodal data, where most graph-based frameworks primarily focus on structural or functional connectivity in isolation, therefore missing out on the complementary information provided by other data types such as genetic, clinical, or cognitive assessments [69,70]. Additionally, the reliance on large amounts of labeled data for training deep graph models remains a hurdle, particularly given the scarcity and heterogeneity of labeled neuroimaging datasets in Alzheimer’s research [71]. These shortcomings highlight the need for more robust, scalable, and interpretable approaches that can effectively integrate diverse data modalities and leverage the rich geometric and relational information inherent in brain imaging data.

Despite these advances, there is limited research on integrating manifold learning and GNNs for Alzheimer’s disease diagnosis. Previous studies have either focused on using manifold learning for visualization and exploration or applied GNNs to predefined brain networks without leveraging the geometric properties of the data. Our work addresses this gap by combining information geometry and manifold learning to construct statistical manifolds from MRI data, which are then represented as graphs for input into GNNs. This approach allows us to capture both the intrinsic geometric structure of the data and the relational information between data points, potentially improving classification accuracy and providing deeper insights into disease progression.

Our study significantly advances the field by demonstrating how information geometry, manifold learning, and graph neural networks can be integrated into a powerful diagnostic framework for classifying Alzheimer’s disease impairment levels. This three-pronged approach leverages the rich geometric structure in MRI data, captures subtle morphological variations through manifold-based modeling, and then processes these relationships via graph neural networks tailored to handle the complex graph-structured outputs. By uniting these advanced techniques, we not only achieve high classification accuracy but also generate interpretable geometrical insights into disease progression. Such a data-driven pipeline can aid clinicians in detecting early pathological changes, refining patient stratification, and monitoring disease trajectories over time. Our work thus points to a robust, scalable methodology that could be extended to other neurodegenerative conditions, ultimately enhancing the precision and reliability of brain imaging–based diagnostics in clinical practice.

## 3. Methodology

### 3.1. Information Geometry

We analyze a dataset D comprising grayscale MRI scans, where each scan is represented as a two-dimensional intensity function $I_{i}:\Omega \to R$. Here, $\Omega \subset R^{2}$ denotes the spatial domain (pixel coordinates), and $I_{i}\left(x,y\right)$ indicates the intensity at position (x,y). The dataset is categorized into *K* distinct classes

(1) $$D=\overset{K}{\underset{k=1}{⋃}}D_{k},$$

where $D_{k}$ consists of $N_{k}$ images belonging to class *k*, each labeled by $y_{i}\in \left\{1,2,\cdots ,K\right\}$.

To manage the high dimensionality of image data, each image is transformed into a lower-dimensional feature vector $x_{i}\in R^{n}$ using a feature extraction mapping Φ, such that

(2) $$x_{i}=\Phi \left(I_{i}\right).$$

Techniques like Principal Component Analysis (PCA) [72,73] are employed for this transformation, leveraging their ability to capture maximum variance in the data efficiently.

We assume that the feature vectors $x_{i}$ are independently and identically distributed (i.i.d.) samples drawn from a class-specific probability distribution $P_{k}\left(x\right)$

(3) $$x_{i}∼P_{k}\left(x\right),\;\mathrm{for}\;y_{i}=k.$$

To model these distributions, we posit that each $P_{k}\left(x\right)$ follows a multivariate Gaussian distribution

(4) $$p_{k}\left(x;\mu_{k},\Sigma_{k}\right)=\frac{1}{{\left(2\pi \right)}^{n/2}{\left|\Sigma_{k}\right|}^{1/2}}\exp\left(-\frac{1}{2}{\left(x-\mu_{k}\right)}^{⊤}\Sigma_{k}^{-1}\left(x-\mu_{k}\right)\right),$$

where $\mu_{k}\in R^{n}$ is the mean vector of class *k*, $\Sigma_{k}\in R^{n\times n}$ is the covariance matrix of class *k*, $|\Sigma_{k}|$ denotes the determinant of $\Sigma_{k}$.

The multivariate Gaussian assumption is widely adopted due to its mathematical tractability and the ease of parameter estimation via Maximum Likelihood Estimation (MLE) [72,73]. However, it may not capture more complex data distributions, such as multimodal or skewed distributions, which can be addressed using alternative methods like Kernel Density Estimation (KDE) [74,75].

The parameters $\mu_{k}$ and $\Sigma_{k}$ are estimated using MLE. For class *k*, the MLE estimates are

(5) $$\begin{array}{cc} \mu_{k} & =\frac{1}{N_{k}}\sum_{i=1}^{N_{k}}x_{i}, \end{array}$$

(6) $$\begin{array}{cc} \Sigma_{k} & =\frac{1}{N_{k}}\sum_{i=1}^{N_{k}}\left(x_{i}-\mu_{k}\right){\left(x_{i}-\mu_{k}\right)}^{⊤}. \end{array}$$

These estimates provide the sample mean and covariance matrix for each class, facilitating subsequent information geometric analysis.

The Fisher Information Matrix (FIM) quantifies the information that the observable data carries about the parameters $\theta_{k}=\left(\mu_{k},\Sigma_{k}\right)$ of the distribution $p_{k}\left(x;\theta_{k}\right)$ [76,77]. For a multivariate Gaussian distribution, the FIM for class *k* is given by

(7) $$I\left(\theta_{k}\right)=\left(\begin{array}{cc} \Sigma_{k}^{-1} & 0\\ 0 & \frac{1}{2}\Sigma_{k}^{-1}⊗\Sigma_{k}^{-1} \end{array}\right),$$

where ⊗ denotes the Kronecker product. This block-diagonal structure indicates that the mean vector and covariance matrix parameters are uncorrelated under the Gaussian assumption.

To assess the dissimilarity between classes, we utilize divergence measures such as the Kullback–Leibler (KL) divergence.

(8) $$D_{\mathrm{KL}}\left(p_{k}∥p_{l}\right)=\int_{R^{n}}p_{k}\left(x;\theta_{k}\right)\ln\frac{p_{k}\left(x;\theta_{k}\right)}{p_{l}\left(x;\theta_{l}\right)}\;dx,$$

which, for multivariate Gaussians, has an analytical form [76]

(9) $$D_{\mathrm{KL}}\left(p_{k}∥p_{l}\right)=\frac{1}{2}\left[\ln\left(\frac{|\Sigma_{l}|}{|\Sigma_{k}|}\right)-n+\mathrm{tr}\left(\Sigma_{l}^{-1}\Sigma_{k}\right)+{\left(\mu_{l}-\mu_{k}\right)}^{⊤}\Sigma_{l}^{-1}\left(\mu_{l}-\mu_{k}\right)\right].$$

The Fisher Information metric induces a Riemannian manifold structure on the space of probability distributions. The geodesic distance $D_{G}$ between two Gaussian distributions $N(\mu_{0},\Sigma_{0})$ and $N(\mu_{1},\Sigma_{1})$ incorporates both mean and covariance differences.

(10) $$D_{G}^{2}={\left(\mu_{1}-\mu_{0}\right)}^{⊤}{\left(\frac{\Sigma_{0}+\Sigma_{1}}{2}\right)}^{-1}\left(\mu_{1}-\mu_{0}\right)+\sum_{i=1}^{n}{\left(\ln\lambda_{i}\right)}^{2},$$

where $\left\{\lambda_{i}\right\}$ are the eigenvalues of $\Sigma_{0}^{-1}\Sigma_{1}$ [76]. This metric provides a comprehensive measure of distributional differences, accounting for both location and dispersion.

Efficient computation of the geodesic distance involves eigenvalue decomposition of $\Sigma_{0}^{-1}\Sigma_{1}$ and matrix inversions, which are well-supported by numerical linear algebra libraries [78]. Ensuring numerical stability and computational efficiency is crucial for handling high-dimensional feature spaces common in MRI data analysis.

The subsequent computational challenge lies in evaluating matrix functions such as square roots and logarithms. These functions are pivotal for accurately determining the geodesic path, especially when dealing with the covariance matrices’ spectral properties. Techniques like spectral decomposition or Schur decomposition are often employed to facilitate these evaluations [78], as they allow for the efficient computation of matrix square roots and logarithms by breaking down the matrices into their constituent eigenvalues and eigenvectors. This decomposition simplifies the complex operations required to transform the covariance matrices along the geodesic path.

### 3.2. Manifold Learning

Building upon the foundation of information geometry, manifold learning serves as a critical component in our framework for analyzing Alzheimer’s Disease MRI data. Information geometry provides a rigorous mathematical structure to model the statistical properties of the data through statistical manifolds, where each manifold corresponds to a distinct class of impairment levels. These manifolds are embedded within a higher-dimensional feature space, capturing both the central tendencies and variances of the underlying probability distributions. To effectively classify and differentiate between these classes, we employ advanced graph-based machine-learning techniques, specifically GNNs, which leverage the geometric insights provided by information geometry to enhance classification performance.

Manifold learning in this context involves representing each class-specific statistical manifold as a graph, where nodes correspond to manifold points, each encapsulating the mean vectors of Gaussian parameters estimated from batches of MRI images, and edges encode the geodesic distances between these points. The geodesic distances, derived from the Fisher Information metric, encapsulate the intrinsic geometric relationships between different statistical distributions, therefore preserving the manifold’s curvature and topology within the graph structure. This graph representation inherently captures both the local and global geometric properties of the data, facilitating the application of GNNs to perform nuanced classification tasks.

Graph Convolutional Networks (GCNs) extend traditional convolutional operations to non-Euclidean data structures, making them particularly suited for our graph-based manifold representations. Mathematically, a GCN layer can be formulated as

(11) $$H^{\left(l+1\right)}=\sigma \left({\tilde{D}}^{-\frac{1}{2}}\tilde{A}{\tilde{D}}^{-\frac{1}{2}}H^{\left(l\right)}W^{\left(l\right)}\right),$$

where $H^{\left(l\right)}\in R^{N\times F_{l}}$ represents the node feature matrix at the (*l*)-th layer, $\tilde{A}=A+I$ is the adjacency matrix augmented with self-loops, $\tilde{D}$ is the diagonal degree matrix of $\tilde{A}$, $W^{\left(l\right)}\in R^{F_{l}\times F_{l+1}}$ is the learnable weight matrix, and σ denotes a nonlinear activation function such as ReLU [79,80]. This operation ensures that each node’s feature vector is updated by aggregating information from its immediate neighbors, therefore capturing local geometric and feature-based relationships within the manifold.

Graph Attention Networks (GATs) introduce an attention mechanism to the convolutional framework, allowing the model to assign varying degrees of importance to different neighboring nodes. This is particularly advantageous in our manifold setting, where certain manifold points may hold more discriminative information for classification purposes. The GAT layer is mathematically defined as

(12) $$H^{\left(l+1\right)}=\sigma \left(\sum_{k=1}^{K}\alpha_{ij}^{\left(k\right)}W^{\left(k\right)}h_{j}^{\left(l\right)}\right),$$

where $\alpha_{ij}^{\left(k\right)}$ represents the attention coefficient for the *k*-th attention head, $W^{\left(k\right)}\in R^{F_{l}\times F_{l+1}}$ is the weight matrix for the *k*-th head, and $h_{j}^{\left(l\right)}$ is the feature vector of the (*j*)-th node at the (*l*)-th layer. The attention coefficients $\alpha_{ij}^{\left(k\right)}$ are computed using a shared attention mechanism, typically involving a learnable weight vector $a\in R^{2F_{l+1}}$ and a nonlinear activation function such as LeakyReLU.

(13) $$e_{ij}=\mathrm{LeakyReLU}\left(a^{⊤}\left[Wh_{i}^{\left(l\right)}\|Wh_{j}^{\left(l\right)}\right]\right),$$

where ‖ denotes concatenation. The attention coefficients are then normalized across all neighbors of node *i* using the SoftMax function

(14) $$\alpha_{ij}^{\left(k\right)}=\frac{\exp(e_{ij}^{\left(k\right)})}{\sum_{j^{\prime }\in N\left(i\right)}\exp\left(e_{ij^{\prime }}^{\left(k\right)}\right)},$$

where N(i) denotes the set of neighboring nodes of node *i* [81,82]. This mechanism enables the GAT to dynamically focus on more relevant parts of the graph, enhancing its ability to discriminate between classes based on the underlying manifold geometry.

GraphSAGE (Graph Sample and Aggregation) offers a scalable approach to inductive representation learning on large graphs by sampling and aggregating features from a node’s local neighborhood. This is particularly beneficial in our application, where the graph sizes can vary depending on the number of manifold points. The GraphSAGE layer can be expressed using a mean aggregator as follows:

(15) $$h_{i}^{\left(l+1\right)}=\sigma \left(W^{\left(l\right)}\cdot \mathrm{MEAN}\left(h_{i}^{\left(l\right)},\left\{h_{j}^{\left(l\right)}|j\in N\left(i\right)\right\}\right)\right),$$

where MEAN denotes the mean aggregator function, and $W^{\left(l\right)}\in R^{2F_{l}\times F_{l+1}}$ is the learnable weight matrix for the *l*-th layer [83]. By aggregating features from sampled neighbors, GraphSAGE effectively captures both the local feature information and the broader graph structure, facilitating robust learning of node representations that reflect the geometric properties of the manifold.

In our framework, GNN models—including GCN, GAT, and GraphSAGE—operate on graph representations constructed from statistical manifolds. The node features, which correspond to the mean vectors of Gaussian parameters, encapsulate the key characteristics of each manifold point. Meanwhile, the adjacency matrices encode geodesic distances, preserving the intrinsic geometry of the manifold. These GNN models iteratively update node representations by aggregating and transforming features based on the graph’s structure, enabling the learning of rich, discriminative embeddings that account for both local and global geometric relationships.

The graph embeddings generated by the GNNs are subsequently aggregated using pooling operations, such as Global Average Pooling, to create graph-level representations suitable for classification tasks. These representations inherently capture the geometric properties of the manifolds, as guided by information geometry, allowing classifiers to effectively distinguish between various Alzheimer’s disease impairment levels. By combining information geometry with advanced manifold learning techniques and GNNs, our approach leverages the interplay between statistical attributes and geometric structures to deliver precise and nuanced classifications of MRI data.

This integration of information geometry and manifold learning through graph-based classifiers not only enhances the models’ ability to discriminate between classes but also provides a deeper understanding of the geometric relationships within the data. GNNs, informed by the Fisher Information Matrix and geodesic distances, are particularly effective at capturing subtle variations and structural complexities within statistical manifolds, enabling more accurate and insightful classifications in the analysis of Alzheimer’s disease.

## 4. Results

### 4.1. Dataset

The dataset evaluated in this research is sourced from the Huggingface Alzheimer’s classification dataset, which is publicly available at https://huggingface.co/datasets/Falah/Alzheimer_MRI (accessed on 14 October 2024). Initially, the dataset comprised a total of 6400 MRI scans distributed across four distinct classes based on the severity of cognitive impairment: 3200 scans for individuals without dementia, 2240 scans for those with very mild dementia, 896 scans for individuals with mild dementia, and 64 scans for those with moderate dementia. The significant imbalance between classes, particularly the markedly lower number of scans in the Mild and Moderate Impairment categories, posed challenges for effective machine-learning model training. To address this imbalance, the Synthetic Minority Over-sampling Technique (SMOTE) [84] is applied to the Mild and Moderate Impairment classes, resulting in an augmented dataset with 2739 scans for Mild Impairment and 2572 scans for Moderate Impairment. Consequently, the final dataset comprised 3200 scans for No Impairment, 3008 scans for Very Mild Impairment, 2739 scans for Mild Impairment, and 2572 scans for Moderate Impairment, ensuring a more balanced distribution across all classes.

In our approach, each resized MRI image is treated as a point on a statistical manifold, enabling the application of information geometry to capture the intricate geometric relationships inherent in the data. To model these relationships effectively, GNNs are employed, where each node in the graph represents a point on the manifold corresponding to an individual MRI scan. The edges between nodes are weighted based on geodesic distances derived from information geometry, specifically using the Fisher Information metric. This weighting scheme reflects the statistical dissimilarities between different impairment classes, allowing the GNN to leverage both local and global geometric structures for accurate classification. The large number of scans in each class ensures robust statistical analyses and enhances the reliability of the machine-learning models by capturing a wide array of anatomical variations and pathological changes associated with Alzheimer’s disease.

### 4.2. Information Geometric Results

This study analyzes grayscale MRI scans categorized into four distinct classes: No Impairment, Very Mild Impairment, Mild Impairment, and Moderate Impairment. Each MRI image is converted to grayscale and uniformly resized to 64×64 pixels to maintain consistency across the dataset. This preprocessing step ensures spatial alignment and eliminates variations arising from scale, rotation, and translation. After preprocessing, the images are flattened into one-dimensional vectors to facilitate efficient computational processing and statistical analysis. To address the high dimensionality inherent in the image data, PCA is employed as a dimensionality reduction technique. PCA is configured to retain components that account for 95% of the variance within the dataset, significantly reducing the feature space while preserving essential information required for robust analysis. This transformation produces lower-dimensional feature vectors, enhancing computational efficiency and mitigating the challenges associated with high-dimensional data.

The dataset is subsequently organized by class to enable class-specific statistical analyses. For each impairment class, the mean vector and covariance matrix of the feature vectors are meticulously estimated. To ensure numerical stability and the invertibility of covariance matrices, which is critical for reliable distance computations, a regularization term of $1\times 10^{-5}$ is added to the diagonal elements of each covariance matrix. This regularization effectively addresses issues related to matrix singularity, ensuring robust estimation of statistical parameters.

**Figure 1:**
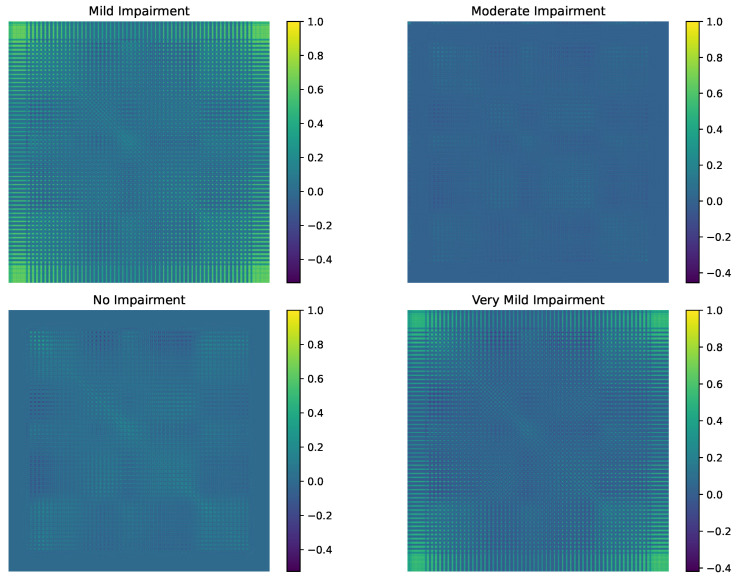
Covariance matrices for impairment classes.

The covariance matrices displayed in Figure 1 provide a visual representation of the interdependencies among features in Alzheimer’s disease impairment classes, ranging from No Impairment to Moderate Impairment. These matrices encapsulate the statistical relationships between individual variables, highlighting patterns of variability and co-variability specific to each impairment class. By normalizing and visualizing the covariance matrices, differences in the structure and intensity of feature correlations across classes become evident, which can be linked to the progression of cognitive decline.

Alzheimer’s disease progressively alters the brain’s functional architecture, as reflected in the covariance matrices of different impairment stages and their corresponding statistical manifold geometries. In the No Impairment class, covariance matrices exhibit low-intensity, uniform correlations, resulting in a smoothly curved manifold that represents a stable and homogeneous brain structure. As impairment begins with the Very Mild stage, subtle increases in feature variability and correlations introduce slight geometric distortions, indicating early cognitive decline and the brain’s adaptive neural plasticity. Progressing to Mild and Moderate Impairment stages, covariance patterns become more heterogeneous and pronounced, leading to significant manifold curvature and complex geodesic paths that signify extensive neuronal loss and disrupted brain organization. These geometric transformations highlight the dynamic impact of Alzheimer’s on brain connectivity and structure.

**Figure 2:**
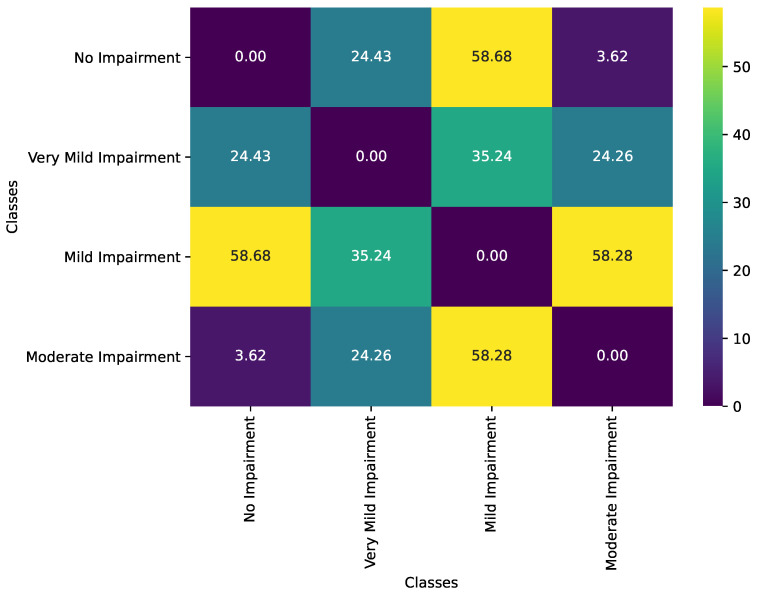
Geodesic distance matrix heat map between impairment classes.

Figure 2 illustrates the geodesic distance matrix between Alzheimer’s disease impairment classes, capturing the statistical dissimilarities between each pair of classes based on their feature distributions. In the context of information geometry, each impairment class is represented as a distinct point on a statistical manifold, with covariance matrices defining the Fisher Information metric that governs the manifold’s geometry. The geodesic distance, computed using this metric, integrates both the mean vectors and covariance matrices of each class, providing a comprehensive measure of dissimilarity that accounts for both location and shape differences in the feature distributions. This matrix effectively highlights the progression of cognitive impairment by quantifying how the underlying feature distributions diverge as impairment levels increase, therefore mapping the trajectory of Alzheimer’s disease progression onto the manifold.

Permutation tests are performed to assess the statistical significance of the computed geodesic distances between each pair of classes. These tests evaluate whether observed geodesic distances are significantly greater than expected under the null hypothesis of no dissimilarity. The test results are presented in Table 1.

The primary objective of this statistical testing is to ascertain whether the observed geodesic distances between pairs of impairment classes were significantly different from what might be expected by random chance. Under the null hypothesis, it is presumed that there is no true dissimilarity between the classes, meaning any observed distance could be attributed to random variations in the data. To test this hypothesis, permutation tests are conducted, a robust non-parametric method ideal for assessing the significance of observed metrics without relying on specific distributional assumptions.

Following the interclass analysis, we examine the intra-class variability, principal geodesic components, and the manifold structure of the Alzheimer’s disease impairment classes using average Mahalanobis distances, Principal Geodesic Analysis (PGA), and Uniform Manifold Approximation and Projection (UMAP), respectively.

**Figure 3:**
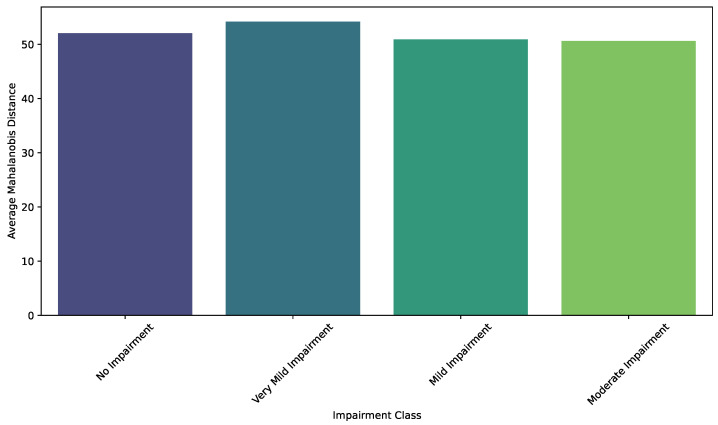
Intra-class variability across impairment classes.

Intra-Class Variability Analysis is quantified using average Mahalanobis distances, which measure the dispersion of individual samples within each impairment class relative to the class mean. Specifically, for each class, the Mahalanobis distance of each sample from the class centroid is calculated using the inverse of the covariance matrix estimated for that class. The average of these distances provides a scalar value representing the overall variability within the class. Figure 3 illustrates these average distances, highlighting the degree of homogeneity or heterogeneity present in each impairment stage.

The bar plot reveals that all classes exhibit substantial variability, indicating the diversity of features within each impairment category. However, differences in the average distances suggest subtle variations in the level of homogeneity among the classes. For instance, the No Impairment and Very Mild Impairment classes demonstrate slightly higher average Mahalanobis distances compared to the Mild Impairment and Moderate Impairment classes. This pattern might indicate a broader feature distribution in the earlier stages of impairment, potentially reflecting greater variability in brain morphology and function in these groups. The higher variability in these classes could be attributed to a wider range of compensatory mechanisms and neurobiological responses that occur during the initial phases of cognitive decline.

**Figure 4:**
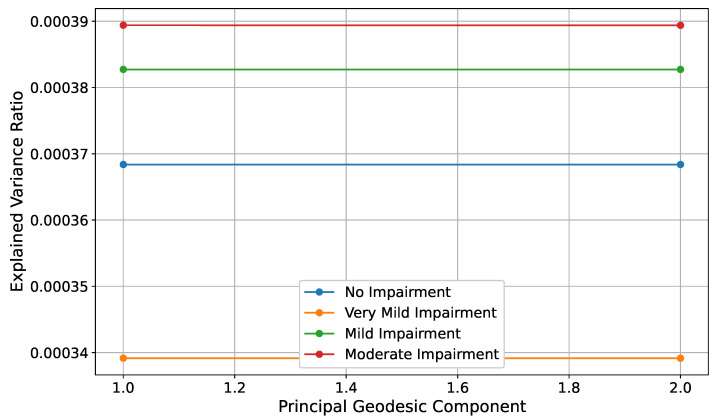
PGA - Explained variables.

PGA is employed to identify the principal modes of variation within each impairment class on the statistical manifold. PGA approximates the principal components in the tangent space at the class mean by first whitening the data using the inverse square root of the covariance matrix. Subsequently, PCA is performed on the transformed data to extract the principal geodesic components. These components capture the directions of maximum variance within the manifold. Figure 4 displays the explained variance ratios for the first two principal geodesic components across the different impairment classes, providing insight into the dominant patterns of variation within each group.

Figure 4 highlights the explained variance ratios for these components, offering a comparative view of the variability within each class. Notably, the explained variance ratios differ slightly across impairment classes, indicating variations in the distribution and complexity of the underlying data.

**Figure 5:**
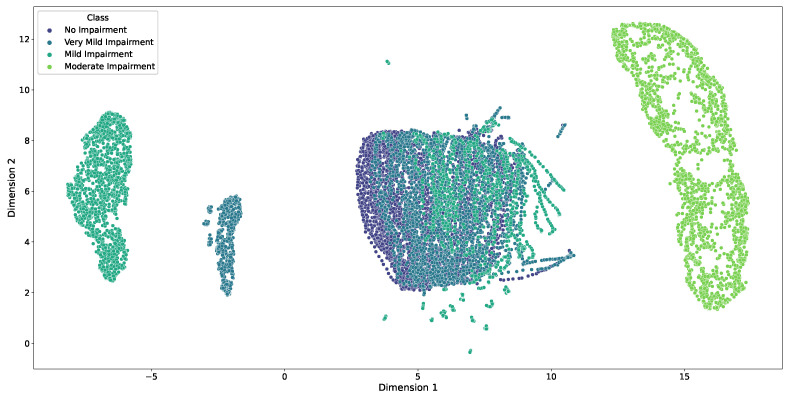
UMAP visualization of impairment classes.

UMAP is utilized to visualize the high-dimensional feature distributions of the impairment classes in a two-dimensional space. UMAP constructs a low-dimensional embedding by preserving both local and global structural relationships from the original data. This technique facilitates the visualization of complex manifolds by mapping similar data points closer together while maintaining the overall topology. Figure 5 presents the UMAP embedding of all samples, with each point color-coded according to its corresponding impairment class. The visualization reveals the spatial arrangement and clustering of the classes within the reduced-dimensional space, offering a comprehensive overview of the feature distribution and class separability.

The No Impairment and Very Mild Impairment classes exhibit substantial overlap, indicating similarity in their feature distributions, which is expected given their close proximity in the clinical spectrum. In contrast, the Mild and Moderate Impairment classes form more distinct clusters, suggesting greater divergence in their feature distributions and potentially reflecting the increased structural and functional changes associated with more severe cognitive decline.

### 4.3. Manifold Learning Results

In the analysis of information geometric results, we construct statistical manifolds by encompassing all MRI images across the different classes. To facilitate manifold learning using Graph Neural Networks (GNNs), it is essential to obtain distinct statistical manifolds within each class. Consequently, we perform sampling of 300 images per class, which are subsequently divided into batches of 10 to estimate Gaussian parameters, specifically the mean and covariance matrices. To ensure numerical stability and prevent singularities, an epsilon value of $1\times 10^{-5}$ is similarly added to each covariance matrix. These estimated parameters define manifold points that serve as the nodes in complete graphs, where each MRI image is represented as a node.

Edges between nodes are weighted based on information-theoretic geodesic distances calculated using the Fisher Information metric. In order to maintain graph connectivity and reduce computational complexity, edges with weights exceeding the 80th percentile or resulting in infinite distances are pruned. This pruning process eliminates weak or unreliable connections that do not contribute significantly to the manifold’s geometry. Subsequently, a minimum spanning tree is enforced to guarantee a connected graph structure, ensuring that all nodes remain accessible within the graph.

By recognizing MRI images as nodes and leveraging geodesic distances for edge weighting, the constructed graphs accurately reflect the underlying geometric relationships inherent in each class’s statistical manifold. This methodological framework provides a robust foundation for manifold learning through GNN architectures, allowing for the effective extraction and analysis of complex patterns within the MRI data. The resulting graphs serve as the input for various GNN models, including GCN, GAT, and GraphSAGE, each designed to capture different aspects of the data’s topology and feature distribution. This comprehensive approach ensures that the manifold learning process is both mathematically rigorous and computationally efficient, ultimately enhancing the classification performance of the GNN models in distinguishing between the different levels of impairment.

The GCN model comprises two GCN layers with 64 hidden units each, incorporating dropout rates of 0.5 to mitigate overfitting. The GAT model consists of two GAT layers; the first layer has 64 units with 8 attention heads, and the second layer has 64 units with a single attention head, both utilizing a dropout rate of 0.6. The GraphSAGE model includes two GraphSAGE layers with 128 hidden units and a dropout rate of 0.6. All models employ the Adam optimizer with a learning rate of $1\times 10^{-4}$ and a gradient clipping norm of 1.0 to ensure stable training dynamics. Training is conducted for a maximum of 100 epochs with early stopping based on validation accuracy, utilizing a batch size of 16.

Feature scaling is performed using StandardScaler, followed by dimensionality reduction through PCA, which reduces the feature dimension to 128 components. This preprocessing step enhances computational efficiency and model performance. The dataset is split into training and testing sets with an 80–20 ratio, ensuring a balanced distribution across classes through stratified sampling. Class weights are computed to address potential imbalances, providing a balanced learning environment for the models.

**Figure 6:**
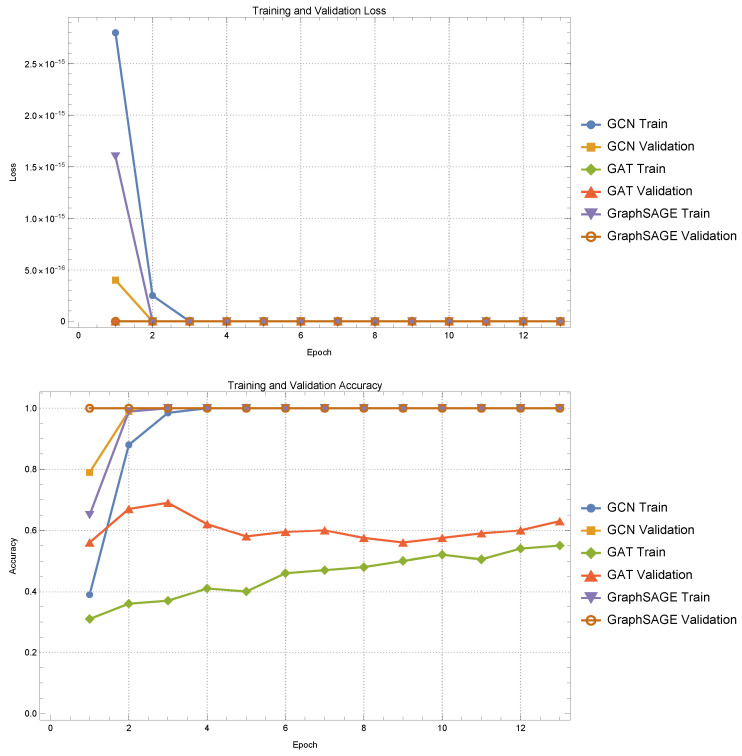
Comparative analysis of GCN, GAT, and GraphSAGE in classification using information-theoretic geodesic graph representations.

Figure 6 illustrates the training and validation performance of three GNN models applied to classification tasks utilizing graph representations of MRI images. The graph construction leverages information-theoretic geodesic distances calculated within statistical manifolds to weight the edges, forming a comprehensive structure that captures the underlying variability and relationships within each class.

**Figure 7:**
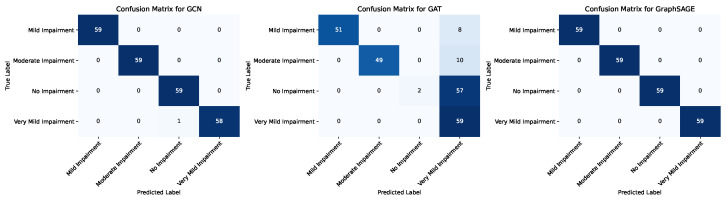
Confusion matrices of GCN, GAT, and GraphSAGE in the classification of different impairment classes.

Figure 7 presents the confusion matrices for three GNN models. These confusion matrices provide a comprehensive evaluation of each model’s capability to accurately classify the four categories of cognitive impairment.

Table 2 delineates the classification performance metrics for three GNNs. The metrics evaluated include Precision, Recall, F1-Score, and Support for each class, alongside overall Accuracy, Macro Average, and Weighted Average scores. Precision for a particular class is defined as the ratio of true positive predictions to the sum of true positive and false positive predictions.

(16) $$\mathrm{Precision}=\frac{TP}{TP+FP}.$$

Recall, also known as sensitivity, is the ratio of true positive predictions to the sum of true positive and false negative predictions

(17) $$\mathrm{Recall}=\frac{TP}{TP+FN}.$$

The F1-Score is the harmonic mean of Precision and Recall, providing a balance between the two metrics

(18) $$F1-\mathrm{Score}=2\times \frac{\mathrm{Precision}\times \mathrm{Recall}}{\mathrm{Precision}+\mathrm{Recall}}.$$

Support is the total number of actual occurrences of the class in the dataset, calculated as the sum of true positives and false negatives

(19) Support=TP+FN.

For both equations, TP represents true positives, FP denotes false positives, and FN signifies false negatives.

## 5. Discussions

### 5.1. Discussions on Information Geometric Results

The covariance matrix for the No Impairment class forms a foundational structure on the statistical manifold, characterized by relatively uniform and low-intensity correlations among features. In the context of information geometry, this class occupies a region of the manifold where the Fisher Information metric reflects a homogeneous and stable brain architecture. This homogeneity signifies that cognitively healthy individuals exhibit minimal variability in their brain features, resulting in a smoothly curved manifold with gentle geodesic paths. The low-intensity correlations indicate that interactions between different brain regions or features are balanced and not overly dependent on one another, allowing the manifold to maintain a stable and resilient geometry. This stable baseline is essential for efficient cognitive functioning and serves as a reference point for detecting deviations as impairment begins to manifest.

Transitioning to the Very Mild Impairment class, the covariance matrices reveal subtle increases in feature variability and correlation intensity, which correspond to a slight distortion in the manifold’s geometry. This stage marks the emergence of early cognitive decline, where the statistical manifold begins to exhibit areas of increased curvature due to the onset of Alzheimer’s-related pathological changes, such as amyloid-beta plaque accumulation and tau tangles. These initial disruptions alter the Fisher Information metric, reflecting changes in the brain’s structural and functional organization. The manifold’s geometry adapts by slightly warping, indicating that the brain is beginning to reorganize its networks in response to emerging impairments. Geodesic paths between the No Impairment and Very Mild Impairment classes become more complex, highlighting the brain’s attempt to compensate for early pathological changes through neural plasticity and adaptive reorganization. This stage is critical for early detection, as the geometric alterations on the manifold can serve as indicators for initiating interventions that may slow or modify the trajectory of cognitive decline.

In the Mild Impairment and Moderate Impairment classes, the covariance matrices depict more pronounced and heterogeneous patterns of variability, significantly altering the manifold’s geometry. The Fisher Information metric in these classes reflects elevated correlation intensities and distinct regions of stronger feature interdependence, signaling a greater degree of disruption in the brain’s organizational structure. In the Mild Impairment stage, the manifold begins to exhibit regions of high curvature and complex geodesic pathways, representing the brain’s strained compensatory mechanisms. These mechanisms lead to increased reliance on certain neural pathways while others deteriorate, resulting in heterogeneous covariance patterns. As the disease progresses to the Moderate Impairment stage, the manifold undergoes further geometric transformation, with more pronounced and widespread curvature changes. This indicates extensive neuronal loss and synaptic dysfunction, which are captured by the covariance matrices through distinct clusters of highly interconnected features. These clusters create localized regions of significant pathology that dominate the overall network dynamics, leading to a more complex and variable geometric structure on the manifold. The transition to the Moderate Impairment class signifies a shift toward more uniform pathological alterations, where the geometry of the manifold becomes consistently disrupted across broader regions, reflecting advanced and widespread cognitive decline.

The evolving differences across the covariance matrices highlight the dynamic impact of Alzheimer’s disease on the functional architecture of the brain, as represented by the statistical manifold. Increasing impairment is mirrored by greater variability and more pronounced correlations between features, which manifest as complex geometric distortions and intricate geodesic paths on the manifold. The observed heterogeneity in the Mild Impairment and Very Mild Impairment classes can be attributed to the transitional nature of these stages, where the brain actively attempts to compensate for early pathological changes through neural plasticity. This compensation introduces variability and complex interdependencies among brain regions, resulting in a manifold that is increasingly curved and intertwined. In contrast, the Moderate Impairment class exhibits a more uniform and severely disrupted geometric structure, where compensatory mechanisms are overwhelmed, and the manifold reflects a stable yet profoundly altered state. This shift from heterogeneous to more uniform disruption underscores the critical phases of Alzheimer’s progression, emphasizing the importance of understanding the manifold’s geometry to identify key intervention points.

Furthermore, the analysis of covariance matrices within the information geometric framework offers valuable insights into potential biomarkers for early detection and monitoring of Alzheimer’s disease. By examining the geometric properties of the manifold, such as curvature and geodesic distances between different impairment classes, researchers and clinicians can identify specific patterns of feature variability and correlation intensities that correspond to various stages of impairment. These geometric signatures can inform the development of targeted diagnostic tools and therapeutic strategies. For example, heightened curvature in particular regions of the manifold during the Very Mild Impairment stage could serve as indicators for initiating preventative treatments. Additionally, understanding the trajectories of geodesic paths in the Mild and Moderate Impairment stages can guide the creation of interventions aimed at stabilizing or restoring functional connectivity, therefore mitigating cognitive decline.

The geodesic distances between the No Impairment class and the other classes reveal a clear progression in statistical dissimilarity, reflecting the geometric transformation of the manifold as cognitive impairment intensifies. The geodesic distance between No Impairment and Very Mild Impairment is moderate (24.43), indicating the onset of subtle cognitive and structural changes that begin to warp the manifold’s geometry. This moderate distance suggests that the Very Mild Impairment class occupies a region of the manifold that is increasingly curved relative to the No Impairment baseline, capturing the initial deviations in feature distributions due to early pathological changes such as amyloid-beta plaque accumulation and tau tangle formation. The spatial separation on the manifold underscores the early stages of network reorganization as the brain begins to compensate for emerging impairments.

As impairment progresses to the Mild Impairment stage, the geodesic distance from the No Impairment class increases significantly to 58.68. This substantial distance reflects a more pronounced divergence in the statistical properties of the feature distributions, corresponding to greater geometric distortion of the manifold. The manifold’s curvature becomes more complex, indicative of extensive neuronal loss and synaptic dysfunction that markedly alter the brain’s structural and functional connectivity. The increased geodesic distance signifies that the Mild Impairment class resides in a region of the manifold that is geometrically distant from the healthy baseline, embodying the advanced disruptions in cognitive processes and brain organization.

Interestingly, the geodesic distance between the No Impairment and Moderate Impairment classes is smaller (3.62) than that between No Impairment and Mild Impairment. This anomaly suggests that the Moderate Impairment class may share certain structural or statistical features with the No Impairment class, potentially due to overlapping feature subsets or limitations in the feature extraction process. Alternatively, it may indicate that the manifold’s geometry at the Moderate Impairment stage converges in specific dimensions, reflecting a plateau in certain pathological changes despite overall cognitive decline.

The relationship between the Very Mild Impairment and Mild Impairment classes, with a geodesic distance of 35.24, captures the intermediate stage of impairment progression. This distance reflects the increasing disruption in feature distributions as the disease advances, manifesting as further geometric deformation of the manifold. Similarly, the geodesic distance between Very Mild Impairment and Moderate Impairment (24.26) is smaller compared to the distance between Mild Impairment and Moderate Impairment (58.28). This pattern underscores the complexity of cognitive decline, where certain stages exhibit overlapping statistical characteristics. Such overlaps may arise from compensatory mechanisms that temporarily stabilize certain network properties or from shared pathological features that affect multiple regions simultaneously, leading to nuanced geometric relationships on the manifold.

The diagonal elements of the matrix are zero, as expected, since they represent the distances of each class to itself, embodying perfect similarity. The off-diagonal elements provide a symmetric view of the pairwise dissimilarities, highlighting how distinct or overlapping the classes are within the statistical manifold. The larger distances between No Impairment and the more clinically severe classes, such as Mild Impairment, emphasize the progressive nature of cognitive decline, as reflected by the increasing geometric separation on the manifold. In contrast, the smaller distances between adjacent classes reflect transitional stages of impairment, where the manifold undergoes gradual yet significant geometric transformations to accommodate the evolving feature distributions.

As a result, the geodesic distance matrix provides a quantitative and geometric depiction of the relationships between Alzheimer’s disease impairment classes within the framework of information geometry. The observed distances align with the expected progression of cognitive decline, capturing both the gradual and distinct shifts in feature distributions as the disease advances. By mapping these impairment classes onto a statistical manifold, the geodesic distances not only quantify the dissimilarities between classes but also reveal the underlying geometric transformations that correspond to neurobiological changes in Alzheimer’s disease.

The permutation test methodology involves several key steps. Initially, feature vectors from two classes are combined into a single dataset. Subsequently, the class labels are randomly shuffled numerous times (e.g., 1000 permutations), and for each permutation, the geodesic distance between the two newly formed groups is recalculated. This process generates a distribution of geodesic distances that would be expected if there are no true differences between the classes. The *p*-value is then determined by calculating the proportion of permuted distances that are equal to or greater than the observed geodesic distance. A *p*-value below a predefined threshold (commonly <0.05) indicates that the observed distance is unlikely to have occurred under the null hypothesis, therefore suggesting a statistically significant difference between the classes.

The results of the permutation tests revealed varying levels of statistical significance across different pairs of impairment classes. Specifically, the geodesic distance between the No Impairment class and the Very Mild Impairment class yields a *p*-value of 0.1020, which is not statistically significant. This suggests that there is insufficient evidence to conclude that these two classes are distinct within the statistical manifold, indicating potential overlap or minimal differentiation in their feature distributions. In contrast, the distance between the No Impairment and Mild Impairment classes is highly significant (*p* = 0.0000), strongly supporting a robust difference in their feature distributions. Similarly, the comparison between Mild Impairment and Moderate Impairment also produced a highly significant *p*-value (*p* = 0.0000), underscoring a clear distinction between these stages of cognitive decline.

Interestingly, the geodesic distance between the No Impairment and Moderate Impairment classes resulted in a *p*-value of 0.8380, indicating a lack of statistical significance. This unexpected result may suggest that these two classes share certain structural or statistical features, possibly due to overlapping feature subsets or limitations inherent in the feature extraction process. Additionally, the distance between the Very Mild Impairment and Moderate Impairment classes (*p* = 0.1010) is not statistically significant, hinting at similarities or transitional characteristics between these stages. Conversely, the distance between Very Mild Impairment and Mild Impairment is statistically significant (*p* = 0.0020), highlighting a meaningful differentiation as cognitive impairment intensifies.

These findings have important implications for understanding the progression of Alzheimer’s disease. The significant *p*-values for distances involving Mild Impairment and Moderate Impairment affirm that these stages are distinctly separable based on their feature distributions, which could reflect substantial neurobiological changes associated with more advanced cognitive decline. On the other hand, the non-significant *p*-values between No Impairment and Very Mild Impairment, as well as between No Impairment and Moderate Impairment, suggest potential overlaps or transitional phases where the distinctions between impairment levels are less clear. This overlap might be attributable to compensatory mechanisms in the brain that temporarily stabilize certain cognitive functions despite underlying pathological changes, or it could indicate that the selected features do not fully capture the nuances between these classes.

The Moderate Impairment class exhibits the highest explained variance for the principal geodesic components, suggesting a more pronounced dominant pattern of variation compared to the other classes. This highly explained variance implies that the majority of the intra-class variability in the Moderate Impairment group can be captured by a single principal geodesic component, reflecting a more structured and possibly more homogeneous alteration in brain features at this advanced stage of cognitive decline. In contrast, the No Impairment and Very Mild Impairment classes show relatively lower explained variance in their principal geodesic components. This lower explained variance indicates that the variability within these groups is distributed more evenly across multiple geodesic directions, suggesting a more uniform or less variable distribution of brain features. The No Impairment class, characterized by cognitively healthy individuals, inherently exhibits minimal variability, which is consistent with the expectation of stable and consistent brain structures in the absence of neurodegenerative processes. Similarly, the Very Mild Impairment class, representing the earliest detectable stage of cognitive decline, displays modest variability that may be attributed to subtle and heterogeneous neurobiological changes as the disease begins to manifest.

The Mild Impairment class demonstrates an intermediate pattern, with explained variance ratios that indicate a moderate level of variability captured by the principal geodesic components. This suggests that while there is a dominant mode of variation within this group, a significant portion of the intra-class variability still spans multiple geodesic directions. This complexity may reflect the onset of more pronounced neurodegenerative changes and the emergence of diverse compensatory mechanisms as cognitive impairment intensifies.

The analysis underscores the utility of PGA in uncovering class-specific patterns of variability that are not readily apparent in the original high-dimensional feature space. By focusing on the principal geodesic components, the figure effectively conveys the extent to which each impairment class is characterized by dominant modes of variation, providing a nuanced understanding of the structural differences and similarities across the classes. The varying explained variance ratios across classes highlight the differential complexity and heterogeneity of brain feature distributions at each stage of Alzheimer’s disease progression. These insights are instrumental in delineating the distinct neurobiological landscapes associated with each impairment stage, therefore enhancing the interpretability of the manifold-based statistical framework employed in this study. Furthermore, the methodological rigor of mapping data to the tangent space and whitening ensures that the PGA accurately captures the intrinsic geometric properties of the statistical manifold. This precision allows for a more faithful representation of the underlying neurobiological variations, facilitating the identification of subtle yet significant patterns of cognitive impairment. The principal geodesic components, therefore, not only serve as indicators of dominant variation directions but also as markers of the evolving complexity of brain structures as Alzheimer’s disease advances.

The UMAP results capture the heterogeneity within the clusters, with some classes displaying tighter groupings and others exhibiting a broader spread of data points. This variability reflects differences in intra-class variability and highlights the nuanced patterns of feature distributions within each impairment level. The preservation of local structures ensures that similar data points remain close in the embedding space, while the preservation of global topology maintains the relative distances between different clusters, aligning with the progression of impairment severity. This dual preservation allows for an accurate representation of both the internal structure of each class and the relationships between classes.

Furthermore, the spatial arrangement of the clusters in the UMAP embedding aligns with the expected trajectory of Alzheimer’s disease progression. The gradual transition from No Impairment to Moderate Impairment is visually evident, with the embedding reflecting the continuum of cognitive decline. The overlapping regions between adjacent classes, such as Very Mild and Mild Impairment, suggest transitional phases where the feature distributions begin to diverge more distinctly, capturing the incremental nature of disease progression. This gradient of separation reinforces the clinical understanding of Alzheimer’s disease as a spectrum rather than a set of discrete categories.

Additionally, the UMAP visualization reveals subtle distinctions within each impairment class that may correspond to underlying biological or cognitive heterogeneities. For instance, within the Moderate Impairment cluster, there may be subgroups that represent different subtypes of neurodegeneration or varying extents of compensatory mechanisms. These internal structures, while not explicitly categorized, provide insights into the complexity and variability of Alzheimer’s disease manifestations.

### 5.2. Discussions on Manifold Learning Results

In the top panel of Figure 6, the training and validation loss trajectories for each model are displayed over the course of 12 epochs. GCN demonstrates rapid convergence within the first few epochs, achieving significantly lower loss values, particularly in the training set, compared to GAT and GraphSAGE. This behavior reflects the model’s ability to effectively propagate and aggregate information from node neighborhoods in the graph. GAT, while showing slower convergence, maintains a consistent and relatively low validation loss, suggesting robust generalization despite its slower training dynamics. GraphSAGE, in contrast, exhibits relatively higher and more fluctuating loss values, particularly in validation, indicative of challenges in effectively aggregating node features or overfitting to training data.

The lower panel of the same figure presents the accuracy trends for training and validation across the same epochs. GCN achieves the highest and most stable accuracy, surpassing both GAT and GraphSAGE, particularly in validation. This highlights GCN’s capacity to learn representations that align well with class distinctions encoded in the statistical manifold-informed graph structure. GAT shows competitive validation accuracy but lags behind in training accuracy, indicating that the attention mechanism in GAT may introduce additional computational overhead that slows convergence but aids in mitigating overfitting. GraphSAGE displays the lowest accuracy overall, reflecting limitations in capturing the nuanced relationships inherent in the data, potentially due to its neighborhood sampling mechanism not fully exploiting the global structure of the statistical manifolds.

The observed trends are intrinsically tied to the methodological framework. This construction emphasizes the importance of the manifold structure in capturing both local and global variability, enabling the models to leverage rich relational information. The distinct behaviors of the models suggest varying sensitivities to the graph’s structure: GCN benefits from its simplicity and efficient aggregation, GAT leverages attention to prioritize important edges at the cost of slower convergence, and GraphSAGE relies on sampling strategies that may overlook critical relationships in densely connected graphs.

The GCN model exhibits outstanding classification performance, as demonstrated by its confusion matrix. The model accurately predicts nearly all samples across the four impairment classes, with only a single misclassification observed in the Very Mild Impairment category, where one instance is incorrectly labeled as No Impairment. This exceptional accuracy underscores the GCN’s effectiveness in leveraging the information-theoretic geodesic graph structure to distinguish between the subtle patterns characterizing each impairment class. The robust feature aggregation mechanism inherent to GCNs significantly contributes to this superior performance.

In contrast, the GAT model demonstrates a modestly higher rate of misclassification, particularly within the Mild Impairment and Moderate Impairment classes. Specifically, eight instances from the Mild Impairment class are incorrectly classified as Very Mild Impairment, and ten samples from the Moderate Impairment class are similarly misclassified. These misclassifications suggest that while the GAT model’s attention mechanism facilitates the capture of detailed relational information, it may introduce complexities that hinder consistent performance across all classes within this dataset. The potential overemphasis on specific graph connections by the attention mechanism could lead to these classification ambiguities.

The GraphSAGE model achieves classification performance comparable to that of the GCN, accurately predicting all samples across each impairment class with no observed misclassifications. This result indicates that GraphSAGE’s neighborhood-based information aggregation effectively captures the structural and relational features encoded within the geodesic-based graph. Notably, this high performance is attained despite GraphSAGE exhibiting less favorable trends in training and validation loss and accuracy metrics, suggesting that its architecture benefits significantly from the inherent structure of the geodesic graph during evaluation.

The primary distinction among the models lies in their architectural approaches to handling the geodesic distance-based graph representations. Both GCN and GraphSAGE demonstrate strong classification performance with minimal errors, indicating their capacity to generalize effectively to the dataset’s characteristics. In contrast, GAT shows slightly inferior performance, likely attributable to its sensitivity to the distribution of edge weights, which may introduce complexities not fully exploited in this classification task. Overall, the results highlight the efficacy of GCN and GraphSAGE in leveraging statistical manifold structures for accurate classification of Alzheimer’s disease impairment levels.

The GCN model exhibits exemplary performance, achieving perfect Precision, Recall, and F1-Score values of 1.0 across all impairment classes. This indicates that the GCN model not only correctly identifies every instance of each class (Recall) but also ensures that all positive predictions are accurate (Precision). Consequently, the F1-Score, which harmonizes Precision and Recall, also reaches an optimal value of 1.0 for each class. The overall Accuracy, Macro Average, and Weighted Average metrics further reinforce the model’s superior classification capabilities, all attaining a perfect score of 1.0. Such flawless performance suggests that the GCN model effectively leverages the underlying geodesic graph structures to discern the nuanced patterns distinguishing each impairment level, highlighting the robustness of its feature aggregation mechanisms in capturing the intricate relationships within the data.

Similarly, the GraphSAGE model mirrors the GCN’s outstanding performance, attaining perfect Precision, Recall, and F1-Score metrics across all classes. This consistency underscores GraphSAGE’s proficiency in aggregating neighborhood information, enabling it to accurately capture and utilize the structural and relational features embedded within the geodesic-based graph representations. The identical performance metrics across both GCN and GraphSAGE indicate that neighborhood sampling and aggregation strategies employed by GraphSAGE are highly effective in this specific application, facilitating precise classification without any misclassifications.

In stark contrast, the GAT model exhibits a markedly different performance profile. While it achieves perfect Precision in the Moderate and Very Mild Impairment classes, its Recall values are substantially lower, particularly for the Mild Impairment (0.4102) and Moderate Impairment (0.3077) classes. The low Recall for these classes indicates that the GAT model fails to identify a significant proportion of actual positive instances, leading to missed classifications. Specifically, eight instances of Mild Impairment are incorrectly classified as Very Mild Impairment, and ten instances of Moderate Impairment are similarly misclassified. Although the GAT model maintains a respectable Precision of 0.8421 for Mild Impairment and perfect Precision for Moderate and Very Mild Impairment, the reduced Recall undermines its overall F1-Score, which averages 0.5968 in the Macro Average and 0.5954 in the Weighted Average. These results suggest that while the attention mechanisms in GAT can capture detailed relationships within the data, they may introduce complexities that hinder the consistent identification of certain impairment levels. The propensity of the attention mechanism to potentially overemphasize specific graph connections could lead to increased classification ambiguities, particularly in classes with overlapping or subtle distinguishing features.

The GCN and GraphSAGE models demonstrate unparalleled classification accuracy, effectively generalizing the dataset’s characteristics without any misclassifications. Their ability to fully leverage the geodesic-based statistical manifold structures facilitates precise differentiation between impairment levels. Conversely, the GAT model, despite its advanced attention mechanisms designed to capture intricate relational information, exhibits limitations in consistently identifying all instances of certain impairment classes. This discrepancy highlights the sensitivity of GAT to the distribution of edge weights and suggests that its architecture may require further refinement to fully exploit the information-theoretic geodesic graph representations. The comparative analysis of these models underscores the efficacy of GCN and GraphSAGE in leveraging manifold-based graph structures for high-precision classification tasks while also indicating potential areas for improvement in attention-based architectures like GAT.

Our findings underscore the remarkable performance of GCN and GraphSAGE, each achieving perfect classification of Alzheimer’s disease impairment levels in our dataset. Although such near-perfect metrics are desirable, they also invite closer scrutiny. For instance, complete accuracy in a complex task like Alzheimer’s disease classification raises concerns about potential overfitting or the limited diversity of our data. A likely contributing factor may be our relatively constrained dataset, which, while carefully curated, may not encompass the full spectrum of structural and functional variability present in real-world Alzheimer’s disease populations. Consequently, caution is warranted in extrapolating these results directly to clinical settings without additional validation. Future work could address this limitation by employing more extensive datasets that include data from multiple centers, varied acquisition protocols, and broader demographic distributions to better capture the heterogeneity of Alzheimer’s disease progression.

In contrast, the GAT model, despite its sophisticated attention mechanism, showed reduced recall in discriminating between Mild Impairment and Moderate Impairment classes. This finding spotlights the possibility that attention-based approaches may sometimes overemphasize certain features at the expense of a balanced perspective on the entire graph. While our results reveal that GAT’s precision remains high, the comparatively lower recall signals that critical aspects of the Mild and Moderate Impairment classes were overlooked. This nuanced interplay between Precision and Recall illustrates the importance of model interpretability and tuning when dealing with subtle disease states. Additional investigations could involve refining GAT hyperparameters or integrating more refined attention strategies that adapt dynamically to class overlaps and manifold geometry.

When juxtaposed with existing studies, our results parallel or exceed commonly reported classification metrics in Alzheimer’s disease research. Traditional machine-learning techniques—such as SVMs, logistic regression, or multi-kernel methods—have often shown accuracies in the 80–98% range for similar classification tasks [41,42]. Deep-learning models, such as CNNs, typically achieve high performance (e.g., over 90% accuracy) by exploiting spatial correlations within MRI scans [43,44]. However, perfect or near-perfect classification remains rare and is typically restricted to small or highly curated datasets. Our manifold-based framework coupled with graph neural networks appears to push these boundaries further, aligning with a few emerging graph-based studies that report comparable or better accuracies for specific datasets [59,60]. Nonetheless, it is essential to emphasize external validation and cross-site replication to ensure that these performance gains are both robust and generalizable. By balancing high accuracy with considerations of overfitting risks, data diversity, and reproducibility, our approach can offer a promising avenue for advancing AD diagnostics while maintaining scientific rigor.

## 6. Conclusions

This study applies information geometry and manifold learning to MRI data for classifying Alzheimer’s disease impairment levels, proposing an approach with substantial diagnostic potential. By transforming MRI scans into statistical manifolds and subsequently examining covariance structures, geodesic distances, and principal geodesic components, we illuminate clear distinctions across four stages: No Impairment, Very Mild, Mild, and Moderate. The findings reveal that increased variability in the covariance matrices—particularly as one progresses from the No Impairment to Moderate classes—correlates with pronounced structural changes in the brain. In parallel, larger geodesic distances between the Mild and Moderate groups suggest that these geometric transformations could be leveraged as quantifiable imaging markers for disease progression. Such insights are valuable for clinicians seeking objective, data-driven tools to detect early pathological changes, stratify patients according to risk, and personalize treatment plans.

Central to our diagnostic framework are graph-based neural networks, specifically GCN and GraphSAGE, both of which achieved near-perfect classification accuracy. This remarkable performance underscores the compatibility of graph neural network architectures with manifold learning in capturing complex relational structures within MRI data. By treating each image representation as a node in a graph, these models effectively utilize local and global connectivity to distinguish subtle shifts in brain features indicative of Alzheimer’s disease. The precise identification of each impairment category suggests that these models can help clinicians detect early changes even when these alterations are nearly indistinguishable to the human eye. Despite potential concerns over overfitting in smaller, preprocessed datasets, our high accuracy rates demonstrate that integrating manifold geometry with sophisticated graph-based learning can significantly improve both the sensitivity and specificity of AD diagnostics.

Looking ahead, several avenues for enhancement could solidify the clinical applicability of this method. First, expanding the dataset to encompass larger and more diverse populations would reinforce the robustness and generalizability of the classification models. Second, the integration of additional imaging techniques—such as functional MRI or PET scans—could provide a more comprehensive overview of both structural and functional changes occurring in the Alzheimer’s disease continuum. Third, optimizing graph neural network architectures, for instance, by refining hyperparameters or incorporating novel attention mechanisms, may further boost classification performance and limit overfitting. Lastly, rigorous external validation through cross-institutional collaborations would lend stronger evidence to the reliability and clinical value of these algorithms. By focusing on these directions, we envision a near-future scenario where manifold learning and GNN-based diagnostics become an integral part of routine workflows, enabling earlier interventions and more personalized treatments for individuals at risk of or living with Alzheimer’s disease.

## Figures and Tables

**Table 1:** Permutation Test Results for Geodesic Distances Between Impairment Classes.

Class Pair	Observed Geodesic Distance	*p*-Value
No Impairment vs. Very Mild Impairment	24.43	0.1020
No Impairment vs. Mild Impairment	58.68	0.0000
No Impairment vs. Moderate Impairment	3.62	0.8380
Very Mild Impairment vs. Mild Impairment	35.24	0.0020
Very Mild Impairment vs. Moderate Impairment	24.26	0.1010
Mild Impairment vs. Moderate Impairment	58.28	0.0000

**Table 2:** Classification metrics for GNN networks.

Model	Class	Precision	Recall	F1-Score	Support
GCN	Mild Impairment	1.0	1.0	1.0	39.0
	Moderate Impairment	1.0	1.0	1.0	39.0
	No Impairment	1.0	1.0	1.0	40.0
	Very Mild Impairment	1.0	1.0	1.0	38.0
	Accuracy			1.0	
	Macro Avg	1.0	1.0	1.0	156.0
	Weighted avg	1.0	1.0	1.0	156.0
GAT	Mild Impairment	0.8421	0.4102	0.5517	39.0
	Moderate Impairment	1.0	0.3077	0.4705	39.0
	No Impairment	0.4	1.0	0.5714	40.0
	Very Mild Impairment	1.0	0.6579	0.7936	38.0
	Accuracy			0.5961	
	Macro Avg	0.8105	0.5939	0.5968	156.0
	Weighted avg	0.8066	0.5961	0.5954	156.0
GraphSAGE	Mild Impairment	1.0	1.0	1.0	39.0
	Moderate Impairment	1.0	1.0	1.0	39.0
	No Impairment	1.0	1.0	1.0	40.0
	Very Mild Impairment	1.0	1.0	1.0	38.0
	Accuracy			1.0	
	Macro Avg	1.0	1.0	1.0	156.0
	Weighted avg	1.0	1.0	1.0	156.0

## Data Availability

The original contributions presented in the study are included in the article. Further inquiries can be directed to the corresponding author.
